# Mr. White, on Hydrocephalus Internus

**Published:** 1800-02

**Authors:** W. White

**Affiliations:** Bath City Dispensary


### *To the Editors of the Medical and Phyfical Journal.*

Gentlemen,

If you think the following cafes and remarks on Hydrocephalus Internus worthy a place in the Medical and Phyfical Journal, it will be efteemed a favour to infert them.

I am, &c.

W. WHITE.

*Bath City Difpenfary, Dec.* 7, 1799.

---

The firft of the cafes alluded to, occurred between four and five years ago; the fubject of it was a boy feven years of age, and of a mufcular, fanguine temperament. His mother informed me, that for a month previous to applying for advice, he had, at intervals, complained of a pain in his head, attended with lofs of appetite and ftrength. Sometimes he vomited, once or twice a day; at other times, once in two or three days; he had alfo fallen down on the floor feveral times in fits; he had been thirfty and feverifh. When I firft faw him, he lay in a ftate of total infenfibility, which had been preceded by a violent, acute pain in his head. On examination, the pupils of his eyes were found much dilated, and did not apparently poffefs the fmalleft degree of irritability. His face was flufhed; his urine difcharged involuntarily, and his bowels were coftive; he made a conftant noife, and his lips were in perpetual convulfive motion. I immediately opened the temporal artery, ordered his head to be fhaved, bathed with vinegar, a blifter to be there applied, and another betwixt his fhoulders. He took fmall dofes of calomel combined with digitalis, and occafionally a draught compofed of Mift. camphor. fpt. æther. vitriol. comp. and tinct. opii. As purgatives produced no effect in ftimulating the inteftines, clyfters were reforted to for that purpofe. After remaining in the ftate above defcribed about a fortnight, evident fymptoms of amendment took place, and he foon recovered his ufual ftrength.

### CASE II.

Aug. 19.—William Sedman, aged nine, of a dark complexion and fpare habit, was feized, three days ago, with a violent pain in his head, attended with vomitting; the latter has ceafed, but the former has continued fo acute as to make him frequently fcream out; at other times he lies in a comatofe ftate: has had feveral convulfion fits. He takes no food, except when a little liquid is forced into his mouth with a tea-fpoon. His pulfe is quick, and fmall; fkin temperate; tongue tolerably clean. When fenfible, complained of thirft; bowels coftive; urine difcharged involuntarily, and copioufly. For a fortnight previous to this attack, his father noticed his being hot at night. On the firft day of his illnefs, an emetic was given, which brought off a great quantity of bile from his ftomach. The following medicines were prefcribed:

℞. Calomel. pp^t. gr. iij. Conferv. rof. q. f. fit. ft. pil. ftatim. fumend.

℞. Magnes. vitriol. ʒij. folve in
Aq. pur. ℥ij. cui add.
Aq. menth. pip. ℥ij. ft. miftur. cujus capiat cochl. j. amplum quartâ quâque horâ.
Applicetur emplaft. Canthar. inter-fcapul.

Aug. 20.—11, A. M. He ftill continues in an infenfible ftate. The pupils are dilated; but at the approach and removal of a lighted candle, they alternately contract and dilate, although he does not take the leaft notice of any of the furrounding objects. Pulfe rather flower than yefterday. Bowels have been moved once.

Applic. hirudines fex temporibus.
Repet. pil. Calomel. ftatim.

℞. Pulv. fol. digital. purpur. gr. ¼.

Calomel. gr. j. Conferv. rof. q. s. ft. pil. mane nocteque fumend.

4, P. M.—Vifited him again, and was informed, that in about ten minutes after the leeches came off, his fenfes returned; he requefted fomething to eat, and made water voluntarily.

Aug. 21.—11, A. M. He continues fenfible, and tolerably free from pain; was reftlefs till about one o'clock in the morning, after which he had four hours found fleep. Pulfe rather quicker than yefterday; has had two motions, but makes very little water.

℞. Kali acetat. ʒj.

Aq. menth. pip.

—— pur. aa ℥ij.

Sumat cochl. j. magn. 4ta. quâque horâ.

8, P. M.—About five o'clock this afternoon he had a febrile acceffion with flight delirium. Has made more water. Complains rather of a pain in his head. Has had no fleep fince the morning. The following draught was prefcribed,

℞. Aq. menth. pip. ʒvi.

Mift. Camphor ʒij.

Tinct. opii. gtt. viij.

Spt. ætheris vitriol. comp. gtt. xx. M.

Aug.

Aug. 22.—Has been reftlefs during the night, although he took the draught. Complains rather of pain in the left fide of his head; at intervals appears flightly delirious; has had one loofe ftool. Had no return of the febrile acceffion yefterday evening.

Applic. iterum hirudines tres tempori finiftro.

Perftet in ufu pil. digital. & miftur.

Aug. 23.—Has had a very good night without an opiate; pain in his head relieved, and remains quite fenfible.

Repet. med.

24.—Continues to mend.

Pergat.

27.—Has now no complaint but debility; and of that he quickly recovered.

### CASE III.

Sept. 10.—Sufannah Harris,* aged 46, of a dark complexion and fpare habit, complained of a pain in her head, chiefly acrofs the *os frontis*, and fometimes at the *occiput*, accompanied with a fenfation of weight. The pain was at intervals fo acute that fhe could fcarce refrain from fcreaming out, and was much increafed when fhe lay in an horizontal pofition. She alfo complained of dimnefs of fight, objects appearing double, and of various colours. She was often fick, and vomited; what fhe threw off tafted four and bitter. Had frequent fhiverings in the courfe of the day, fucceeded by hot flufhings; fhe had alfo great anxiety and reftlefsnefs. Her pulfe was hard and frequent, but had not a full ftroke; fkin warm; tongue white, although fhe had not much thirft; her bowels were regular, and fhe made a proper quantity of water. Her appetite was not very good, and fhe refted badly at night. Several of her fingers were clenched, and fhe had a great deal of convulfive motion. The catamenia were upon her. She had been ill about three weeks, and was at firft feized with an head-ach and chillnefs.

Mittatur fang. ad ℥xij.

℞. Calomelas gr. vj.

Pulv. Jalap. gr. viij. ft. bol. mane fumend.

Sumat miftur. falin. ℥j. 4ta quaque horâ.

The blood was exceedingly fizy, affuming the cup-like appearance.

11.—The pain in her head was much abated; pulfe not fo hard,

* I would obferve in this place, that, on inquiry, I found fhe had received a blow on the head, fome time previous to her illnefs.

hard, and lefs frequent; fkin cool; tongue cleaner. Her fingers were not fo much contracted, and her fight was rather better. The bolus had opened her bowels fufficiently. She was ordered to continue the mixture.

12.—Her head was rather more painful than yefterday. Pulfe fomewhat increafed in hardnefs and frequency.

Admoveantur hirudines fex temporibus, et imponatur Empl. Canthar. inter fcapul.

Contin. mift. falin.

13.—Her head was much better, and the anxiety and reftleffnefs confiderably abated. Pulfe lefs hard and frequent. She had quite recovered the ufe of her fingers. Her bowels were open.

Repet. miftur.

15.—Her head free from pain, but the dimnefs of fight not entirely removed.

Repet. miftur.

18.—Head ftill continued eafy. Pulfe fmall and weak, but by no means quick. Her reft at night was much better. She had ftill flight febrile alternations.

Repet. miftur.

25.—Had no return of pain in her head, and felt no complaint but debility, on which account a cordial mixture was prefcribed.

I am extremely forry to add, that in the courfe of ten days I was again requefted to vifit her, although at the laft report fhe was able to attend at the Difpenfary herfelf. She now complained of a violent pain in her ftomach, accompanied with ficknefs and vomiting; her pulfe, as at firft, was hard and frequent. As the fymptoms indicated a re-commencement of inflammatory action, eight ounces of blood were taken from her arm, which exhibited the fame appearance as before. As her head feemed but little affected, I apprehended there might be a metaftafis of the inflammation to the ftomach; to which part a blifter was applied, and a faline draught in the effervefcing ftate ordered to be given every four hours. By thefe means her ftomach was relieved from the violence of the pain, but the ficknefs and vomiting ftill continued. There was no evacuation from the bowels, unlefs by means of purgatives. Her head became again more affected with pain, particularly in the left fide, and was fometimes violently agitated with convulfive motions. The pupils were more than ufually contracted, and flightly fenfible to the ftimulus of light, fo that fhe could difcern objects, though but confufedly. Her pulfe became preternaturally flow. She was perfectly fenfible.

Notwithftanding the application of leeches to her temples, and and a blifter to her head, with various other remedies, fhe died in the courfe of a fortnight.

On examining the head, the right lateral ventricle was quite empty, but the left lateral one contained about half an ounce of clear fluid. Neither the veffels of the *dura* or *pia mater* were turgid with blood; the finufes were alfo empty. No other morbid appearance was obferved.

---

The two laft cafes, I think, clearly demonftrate the great utility of evacuation principally; and if this propofition be granted, it will naturally follow, that where evacuation is proper, ftimulants muft be the reverfe. It is true, fome medicines of alleged ftimulant power were exhibited; but that was chiefly with a view to palliate certain fymptoms, and then, not till after evacuants had been employed.

In this difeafe, we have not only to lament the frequent infidious and equivocal nature of its fymptoms, but alfo the oppofite opinions that are ftill entertained by medical writers, refpecting its nature or proximate caufe, which muft prove an additional embarraffment to the young practitioner.

Dr. Whytt, to whom we are greatly indebted for a very minute defcription of the fymptoms ufually attendant on the difeafe, obferves, "The immediate caufe of every kind of dropfy is the fame, viz. fuch a ftate of the parts as makes the exhalent arteries throw out a greater quantity of fluids than the abforbents can take up." Which ftate, from what he afterwards mentions, he evidently confidered as confifting in debility.

Dr. Darwin thinks inactivity, or torpor of the abforbent veffels of the brain, is the caufe of *hydrocephalus internus*; yet, in another part of his work, he acknowledges, that the torpor of the abforbent veffels may frequently exift as a fecondary effect.

Mr. Brown fays, "that hydrocephalus internus is evidently a difeafe of debility, and requires remedies which increafe the excitability of the fyftem."

There are likewife others who view the fubject in a very different light; and, I think more properly. Dr. Withering obferves, that in very many cafes, if not in all, congeftion or flight inflammation, are the præcurfors to the aqueous accumulation.

Dr. Rufh is of opinion, that inftead of being confidered as an idiopathic dropfy, it fhould be confidered only as an effect of a primary inflammation, or congeftion of blood in the brain. "It appears, (fays he) that the difeafe, in its firft ftage, is the effect fect of caufes which produce a lefs degree of that inflammation which conftitutes phrenitis ; and, that its fecond ftage is a lefs degree of that effufion which produces ferous apoplexy in adults. The former partakes of the nature of the chronic inflammation of Dr. Cullen, and the afthenic inflammation or Dr. Brown." I have taken the liberty (he farther adds) to call it phrenicula, from its being a diminutive fpecies or ftate of phrenitis.

Dr. Beddoes fays, he believes it to belong to inflammations, and that, at an early period of the difeafe, he fhould be tempted to bleed as largely as in pneumonia.

This difference of opinions, may, in fome degree, be accounted for, if we advert to the divifion of the difeafe into the acute and chronic fpecies. "In the former, the difeafe generally proves fatal in lefs than a month; and it is feldom that more than two or three ounces of blood are found within the ventricles. In the latter fpecies, the patient furvives for many months; fometimes for a year or two; and the bones of the cranium are feparated from each other to a great diftance."

From a variety of other cafes of hydrocephalus internus, as well as the preceding, which have fallen under my own obfervation, I am led to think, that the difeafe very rarely occurs from mere debility, as a primary caufe; but that debility is an effect induced by a previous excefs of action in the arterial fyftem.

The great analogy fubfifting between the fymptoms which are characteriftic of inflammation, and thofe which form the firft ftage of the acute fpecies of hydrocephalus internus, together with the good effects often confequent on blood-letting, and the inflammatory appearance which the blood frequently exhibits, are, I think, ftrong proofs of the difeafe being an active inflammation. It is very obvious, that the firft ftage of hydrocephalus internus is attended with an augmentation of the fenfibility of the brain, from the violent acute pain of that organ; the painful fenfation experienced on the eyes being expofed to the ftimulus of light; and the fimilar painful effect produced by noife on the organ of hearing; which phænomena are confidered by nofological writers, as pathognomonic fymptoms of inflammation.* Dr. Darwin fays, that when the eye is inflamed, light becomes eminently painful, owing to the increafed irritative motions of the retina, and the confequent increafed fenfation. Dr. Rufh alfo obferves, that he has remarked an uncommon acutenefs in hearing attend two cafes of

* A legitimate inflammation is always attended with a painful fenfibility in the nerves.—Pearfon's Principles of Surgery, p. 5.

of this diſorder: in one of them, the ſparks which were diſcharged from an hiccory fire, produced great pain and ſtartings, which threatened convulſions.

The termination of inflammation by effuſion into the other cavities, is an additional proof of the preceding ſuggeſtions, reſpecting the nature of the diſeaſe; as there are inſtances of pneumonic inflammation terminating in hydrothorax.

The deſcription which has been given by Dr. Geapengieſier of the hydrops plethoricus, ſtill further confirms what I have ſtated, relative to the nature of hydrocephalus. He obſerves, "in the diſſection of thoſe dying of this diſeaſe, (hydrops plethoricus) collections of water have been found over the whole body; but particularly in the cavities of the thorax and cranium, the large veſſels have been found turgid, being diſtended with much blood. When the diſeaſe has been of the acute kind, no inconſiderable inflammations have been obſerved.

For a more particular account of this ſpecies of dropſy, I would refer to the Fourth Volume of the Medical and Chirurgical Review.

With regard to the curative indications of this diſeaſe, the neceſſity of blood-letting cannot, I think, be too forcibly impreſſed upon the minds of young practitioners; and it ought to be carried to ſuch an extent, as to anſwer a determinate end, viz. that of leſſening topical congeſtion, and diminiſhing arterial action. Although the primary ſtage is the only period in which we can reaſonably hope to derive any advantage from this remedy; yet, even when the ſymptoms which characterize the ſecond ſtage have commenced, I would at leaſt adviſe a topical bleeding; as there is reaſon to apprehend, that the ſame effect is ſometimes produced from mere diſtention of the veſſels, as when effuſion has taken place. The ſudden relief ſometimes obtained by bleeding, I think, favours this idea.—In ſhort, I conſider what Dr. Beddoes has ſaid, reſpecting veneſection, to be of more practical utility than all that has hitherto been written on the ſubject.

Purging is alſo neceſſary, not only on account of leſſening determination to the head, but particularly as the ſymptoms, which proceed merely from foulneſs in the ſtomach and bowels, reſemble thoſe of hydrocephalus, and have been frequently ſoon removed by evacuating the bowels;—caſes of this kind are recorded by Dr. Armſtrong and Dr. Underwood.

Bliſters appear alſo proper; and Dr. Ruſh obſerves, that they ſhould be omitted in no ſtage of the diſorder; for even in the inflammatory ſtage, the diſcharge they occaſion from the veſſels of the head, greatly overbalances their ſtimulating effects upon the whole ſyſtem: lately, it has been recommended to apply them them in the courfe of the futures, and to keep up a difcharge by means of an iffue; but as the *ceratum fabinæ* is capable of exciting a proper difcharging furface, it appears preferable, its application being much lefs troublefome than that of an iffue.

I have frequently given the *digitalis*; and I think with Dr. Farrier, that it appears adapted to fome indication in every fpecies, and every ftage of the diforder; as, promoting abforption, leffening irritation, and diminifhing fever.

With regard to ftimulants, in the firft ftage of this difeafe, when there is evidently increafed action with local congeftion, they muft obvioufly do harm; for in that cafe, as Dr. Darwin judicioufly obferves, they increafe the action of the fecerning, more than of the abforbent fyftem; but, after copious evacuations, the refiftance to the progrefs of the abforbed fluids is removed, and ftimulants then applied, increafe the action of the abforbent fyftem, more than that of the fecerning one.

This obfervation is exemplified in fcrophulous ophthalmia, where we find the complaint frequently increafed by the application of ftimulants, if ufed previous to the removal of the local plethora, by topical bleeding.

Much has been faid in favour of mercury in this difeafe; but I muft confefs, that I never faw any good effects refulting from its exhibition, uncombined with digitalis.

